# Two new species of Neotropical *Lepidocyrtus* Bourlet, 1839 (Collembola, Entomobryidae, Lepidocyrtinae) from Colombia

**DOI:** 10.3897/BDJ.14.e207059

**Published:** 2026-10-08

**Authors:** Luis Antonio González Montaña

**Affiliations:** 1 Museo de Historia Natural Unillanos, Universidad de los Llanos, Villavicencio, Colombia Museo de Historia Natural Unillanos, Universidad de los Llanos Villavicencio Colombia

**Keywords:** Antomical ontology, Neotropical Region, *
Setogaster
*, springtails, taxonomy

## Abstract

**Background:**

The taxonomic revision of Neotropical *Lepidocyrtus*, based on morphological data of the Neotropical species of the genus *Lepidocyrtus*, is currently in progress, which has led to the discovery of two new species for Colombia: Lepidocyrtus (Setogaster) deiverandersoni **sp. nov**., defined mainly by supplementary chaeta ml absent on Abd. II, chaeta p7 as mic on Abd. III and dorsal mac formula A_0_/00/0100+3 and *Lepidocyrtus rosanae* **sp. nov**., defined mainly by labral chaetae a1 branched apically, appendages absent on sublobal plate, sens as absent on Abd. II, sens as and ms absent on Abd. III and dorsal mac formula 00/0100+2. *Lepidocyrtus* (*S.* ) *deiverandersoni* **sp. nov**. ia placed in *Setogaster* is based on the absence of the apical bulb on Ant. IV and mucronal spinelet, while the subgeneric position of *L. rosanae* **sp. nov**. is not clear due to the presence of the apical bulb on Ant. IV and presence of scales on the anterior side of the ventral tube.

**New information:**

Two new species are described from Colombia, Lepidocyrtus (Setogaster) deiverandersoni **sp. nov**. and *Lepidocyrtus rosanae* **sp. nov**., based on body chaetotaxy.

## Introduction

*Lepidocyrtus* Bourlet, 1839 is one of the largest genera of Entomobryidae with approximately 289 described species (Bellinger et al. 2024). However, the true species richness within Neotropical Region may be underestimated (Enguídanos et al. 2024). For Colombia, Mari Mutt (1987) described *L. nilatus* Mari Mutt, 1987, from the south-western part of the country and also recorded *L. nigrosetosus* Folsom, 1927, a species previously known from Puerto Rico and Jamaica (Folsom 1927, Mari Mutt 1987). Cipola et al. (2019) described L. (Fractocyrtus) mateosi Cipola & Bellini, 2019 from Colombian Amazon and, recently, Cipola and Viana (2023) added L. (Fractocyrtus) andensis Cipola & Viana, 2023 from the western Andes of Colombia. The present study aims to describe two new species from the eastern Andes, contributing to the knowledge of *Lepidocyrtus* fauna in Colombia.

## Materials and methods

Samples were collected using pitfall and malaise traps from two localities on Eastern Andes Mountain of Colombia. For morphological examination, the speciemens were cleared with Nesbitt's solution and slide-mounted with Hoyer’s medium. The drawings were made with a drawing tube attached to NIKON E400 and NIKON ECLIPSE E600 phase-contrast microscopes. For the dorsal cephalic chaetotaxy, the "AMS" nomenclature system follows (Soto-Adames 2010); for the labial chaetotaxy, Gisin (1964); for labial papilla, Fjellberg (1999); for postlabial chaetotaxy, Chen and Christiansen (1993); for dorsal macrochaetotaxy formula, Gisin (1967); for dorsal chaetotaxy of thoracic and abdominal tergite, Szeptycki (1979), Zhang et al. (2019), and Mateos et al. (2021); and for distribution of pseudopores, Mateos et al. (2021). Most of the anatomical and phenotypic terms used were taken from the Collembola Anatomy Ontology (CLAO), which is a database with anatomical terms employed in morphological descriptions of Collembola (González Montaña et al. 2023) and Phenotype And Trait Ontology (PATO) (Gkoutos et al. 2004). List of anatomical and phenotypic terms are referred to in the Suppl. material 1. Acronyms show differences between CLAO and natural language descriptions; for example, cephalic chaeta A0 with chaeta A0.h because chaeta A0 also refers to other body chaetae. Anatomical terms are available at https://github.com/luis-gonzalez-m/Collembola.

**Abbreviations**. The following abbreviations are used in this study: Th. II – mesothorax; Th. III – metathorax; Abd. I–VI – abdominal segment I–VI; Ant. I–IV – antennal segment; mac – macrochaeta (-ae); mes – mesochaeta (-ae); mic – microchaeta (-ae); sens – S-chaeta (-ae); BT – basal ungual tooth; MT – median ungual tooth; DT – distal ungual tooth; cx – coxae. Symbols and types of chaetae used to illustrate the chaetotaxy are presented in Fig. 1.

Type material will be deposited at the Museo Entomológico Facultad de Agronomía (UNAB) in Bogotá (Colombia) and at Museo de Historia Natural Unillanos (MHNU-E) in Villavicencio (Colombia).

## Taxon treatments

### Lepidocyrtus (Setogaster) deiverandersoni

González-Montaña sp. nov.

DDCA0401-1654-505B-9AA9-2C80EC812995

33D4EF46-BBC6-420C-A89C-82CB83FE05FB

#### Materials

- **Type status:** Holotype. **Occurrence:** recordedBy: L. A. González-Montaña; individualCount: 1; sex: female; occurrenceID: DA4F9314-8DD8-5B6D-BB19-0B435F80C49C; **Taxon:** genus: Lepidocyrtus; specificEpithet: deiverandersoni; scientificNameAuthorship: González-Montaña, L. A. ; **Location:** country: Colombia; stateProvince: Boyacá; county: Colombia; locality: Santana; verbatimElevation: 2200 m; verbatimLatitude: 6°01’44.9”N; verbatimLongitude: 73°28’57.0”E; **Event:** year: 2018; month: 4; day: 5
- **Type status:** Paratype. **Occurrence:** recordedBy: L. A. González-Montaña; individualCount: 1; occurrenceID: 362EFA7A-1B31-5ECE-B041-4AD4D5D76735; **Taxon:** genus: Lepidocyrtus; specificEpithet: deiverandersoni; scientificNameAuthorship: González-Montaña, L. A. ; **Location:** country: Colombia; stateProvince: Boyacá; county: Colombia; locality: Santana; verbatimElevation: 2200 m; verbatimLatitude: 6°01’44.9”N; verbatimLongitude: 73°28’57.0”E; **Event:** year: 2018; month: 4; day: 5
- **Type status:** Paratype. **Occurrence:** recordedBy: L. A. González-Montaña; individualCount: 1; occurrenceID: EF1402EB-B386-5790-9C21-5AB4CFD28231; **Taxon:** genus: Lepidocyrtus; specificEpithet: deiverandersoni; scientificNameAuthorship: González-Montaña, L. A. ; **Location:** country: Colombia; stateProvince: Boyacá; county: Colombia; locality: Santana; verbatimElevation: 2200 m; verbatimLatitude: 6°01’44.9”N; verbatimLongitude: 73°28’57.0”E; **Event:** year: 2018; month: 4; day: 5
- **Type status:** Paratype. **Occurrence:** recordedBy: L. A. González-Montaña; individualCount: 1; occurrenceID: 45D025BC-C9D6-5D1E-B12A-2D118E1580AE; **Taxon:** genus: Lepidocyrtus; specificEpithet: deiverandersoni; scientificNameAuthorship: González-Montaña, L. A. ; **Location:** country: Colombia; stateProvince: Boyacá; county: Colombia; locality: Santana; verbatimElevation: 2200 m; verbatimLatitude: 6°01’44.9”N; verbatimLongitude: 73°28’57.0”E; **Event:** year: 2018; month: 4; day: 5
- **Type status:** Paratype. **Occurrence:** recordedBy: L. A. González-Montaña; individualCount: 1; occurrenceID: 06A46199-3AAE-556E-909B-A4BE6A80EA3F; **Taxon:** genus: Lepidocyrtus; specificEpithet: deiverandersoni; scientificNameAuthorship: González-Montaña, L. A. ; **Location:** country: Colombia; stateProvince: Boyacá; county: Colombia; locality: Santana; verbatimElevation: 2200 m; verbatimLatitude: 6°01’44.9”N; verbatimLongitude: 73°28’57.0”E; **Event:** year: 2018; month: 4; day: 5
- **Type status:** Paratype. **Occurrence:** recordedBy: L. A. González-Montaña; individualCount: 1; occurrenceID: D0346A07-DCD1-584B-A147-882A5CCEF431; **Taxon:** genus: Lepidocyrtus; specificEpithet: deiverandersoni; scientificNameAuthorship: González-Montaña, L. A. ; **Location:** country: Colombia; stateProvince: Boyacá; county: Colombia; locality: Santana; verbatimElevation: 2200 m; verbatimLatitude: 6°01’44.9”N; verbatimLongitude: 73°28’57.0”E; **Event:** year: 2018; month: 4; day: 5
- **Type status:** Paratype. **Occurrence:** recordedBy: L. A. González-Montaña; individualCount: 1; occurrenceID: AEB5CB1E-5C3E-5CD8-B0DB-C206B5ECE502; **Taxon:** genus: Lepidocyrtus; specificEpithet: deiverandersoni; scientificNameAuthorship: González-Montaña, L. A. ; **Location:** country: Colombia; stateProvince: Boyacá; county: Colombia; locality: Santana; verbatimElevation: 2200 m; verbatimLatitude: 6°01’44.9”N; verbatimLongitude: 73°28’57.0”E; **Event:** year: 2018; month: 4; day: 5
- **Type status:** Paratype. **Occurrence:** recordedBy: L. A. González-Montaña; individualCount: 1; occurrenceID: B72A84A2-4E9E-51A5-982F-2EA93CEA1D38; **Taxon:** genus: Lepidocyrtus; specificEpithet: deiverandersoni; scientificNameAuthorship: González-Montaña, L. A. ; **Location:** country: Colombia; stateProvince: Boyacá; county: Colombia; locality: Santana; verbatimElevation: 2200 m; verbatimLatitude: 6°01’44.9”N; verbatimLongitude: 73°28’57.0”E; **Event:** year: 2018; month: 4; day: 5
- **Type status:** Paratype. **Occurrence:** recordedBy: L. A. González-Montaña; individualCount: 1; occurrenceID: 067A580B-4512-5263-A444-9DE420DC5A0E; **Taxon:** genus: Lepidocyrtus; specificEpithet: deiverandersoni; scientificNameAuthorship: González-Montaña, L. A. ; **Location:** country: Colombia; stateProvince: Boyacá; county: Colombia; locality: Santana; verbatimElevation: 2200 m; verbatimLatitude: 6°01’44.9”N; verbatimLongitude: 73°28’57.0”E; **Event:** year: 2018; month: 4; day: 5
- **Type status:** Paratype. **Occurrence:** recordedBy: L. A. González-Montaña; individualCount: 1; occurrenceID: 349A3016-692B-5014-B003-85DEB7530D32; **Taxon:** genus: Lepidocyrtus; specificEpithet: deiverandersoni; scientificNameAuthorship: González-Montaña, L. A. ; **Location:** country: Colombia; stateProvince: Boyacá; county: Colombia; locality: Santana; verbatimElevation: 2200 m; verbatimLatitude: 6°01’44.9”N; verbatimLongitude: 73°28’57.0”E; **Event:** year: 2018; month: 4; day: 5
- **Type status:** Paratype. **Occurrence:** recordedBy: L. A. González-Montaña; individualCount: 1; occurrenceID: 1F800A1E-8294-5C5C-8C96-A866CEA515BF; **Taxon:** genus: Lepidocyrtus; specificEpithet: deiverandersoni; scientificNameAuthorship: González-Montaña, L. A. ; **Location:** country: Colombia; stateProvince: Boyacá; county: Colombia; locality: Santana; verbatimElevation: 2200 m; verbatimLatitude: 6°01’44.9”N; verbatimLongitude: 73°28’57.0”E; **Event:** year: 2018; month: 4; day: 5
- **Type status:** Paratype. **Occurrence:** recordedBy: L. A. González-Montaña; individualCount: 1; occurrenceID: EB252295-26DC-52FE-80CB-F38A431829FD; **Taxon:** genus: Lepidocyrtus; specificEpithet: deiverandersoni; scientificNameAuthorship: González-Montaña, L. A. ; **Location:** country: Colombia; stateProvince: Boyacá; county: Colombia; locality: Santana; verbatimElevation: 2200 m; verbatimLatitude: 6°01’44.9”N; verbatimLongitude: 73°28’57.0”E; **Event:** year: 2018; month: 4; day: 5
- **Type status:** Paratype. **Occurrence:** recordedBy: L. A. González-Montaña; individualCount: 1; occurrenceID: 627DBEFB-64F0-5A3E-9AD8-A146423CB940; **Taxon:** genus: Lepidocyrtus; specificEpithet: deiverandersoni; scientificNameAuthorship: González-Montaña, L. A. ; **Location:** country: Colombia; stateProvince: Boyacá; county: Colombia; locality: Santana; verbatimElevation: 2200 m; verbatimLatitude: 6°01’44.9”N; verbatimLongitude: 73°28’57.0”E; **Event:** year: 2018; month: 4; day: 5
- **Type status:** Paratype. **Occurrence:** recordedBy: L. A. González-Montaña; individualCount: 1; occurrenceID: 455EF2BE-0AFB-58DF-B812-7269EF019466; **Taxon:** genus: Lepidocyrtus; specificEpithet: deiverandersoni; scientificNameAuthorship: González-Montaña, L. A. ; **Location:** country: Colombia; stateProvince: Boyacá; county: Colombia; locality: Santana; verbatimElevation: 2200 m; verbatimLatitude: 6°01’44.9”N; verbatimLongitude: 73°28’57.0”E; **Event:** year: 2018; month: 4; day: 5
- **Type status:** Paratype. **Occurrence:** recordedBy: L. A. González-Montaña; individualCount: 1; occurrenceID: 296B2979-B15A-5153-BE9A-FD08AA70A9EE; **Taxon:** genus: Lepidocyrtus; specificEpithet: deiverandersoni; scientificNameAuthorship: González-Montaña, L. A. ; **Location:** country: Colombia; stateProvince: Boyacá; county: Colombia; locality: Santana; verbatimElevation: 2200 m; verbatimLatitude: 6°01’44.9”N; verbatimLongitude: 73°28’57.0”E; **Event:** year: 2018; month: 4; day: 5
- **Type status:** Paratype. **Occurrence:** recordedBy: L. A. González-Montaña; individualCount: 1; occurrenceID: 9D85AA83-5188-5CC2-A8C0-43A8C3468527; **Taxon:** genus: Lepidocyrtus; specificEpithet: deiverandersoni; scientificNameAuthorship: González-Montaña, L. A. ; **Location:** country: Colombia; stateProvince: Boyacá; county: Colombia; locality: Santana; verbatimElevation: 2200 m; verbatimLatitude: 6°01’44.9”N; verbatimLongitude: 73°28’57.0”E; **Event:** year: 2018; month: 4; day: 5

#### Description

Holotype body length without appendages 1.69 mm, mean length 1.37 mm (n = 16). Antennae with dark blue colour. Th. II not protruding over head. Th. II with background whitish and diffuse blue on the lateral sides. Th. III to Abd. III with dark blue colour. Abd. IV with background whitish and dark blue spot on posterolateral side (Fig. 2A). Body colour variation is observed with background diffuse blue on Th. II, mediodorsal band dark blue on Abd. IV and coxae with dark blue colour (Fig. 2B).

**Head**. Dorsal cephalic chaetae with A0 as mac, A1–2, A2a as mes, chaetae An on head between 1–14, other chaetae as mic (Fig. 3A). Interocular chaetae q, s, p, r, t present and intraocular scales absent.

Clypeal chaetae ciliate. Prelabral chaetae ciliate. Labral chaetae a1–2, m0–2 and p0–2 smooth; four labral papillae with inner papillae apically rounded and outer papillae apically truncate.

Maxillary outer lobe with the apical and basal chaeta smooth and the sublobal plate with three appendages.

Labial chaetae a1–5 smooth and posterior row with formula M_2_EL_1_L_2_, chaetae ciliated with the same length, r absent (Fig. 3B). Labial papillae A–E and one hypostomal papilla (H) with 0, 5, 0, 3, 4, 2 guard appendages, labial papilla E with lateral process with finger-shape and not reaching tip of papilla (Fig. 3C). Postlabial chaetotaxy with all chaetae ciliated, five chaetae along ventral groove, lateral spine-like chaeta present and three rows with 4 (G1– G4), 1 (X3) and 3 (H1–H3).

**Body**. Dorsal macrochaetae formula 00/0100+3. Th. II as in Fig. 3D. Th. III sens al not observed, other chaetae as in Fig. 3E. Chaetotaxy of Abd. I as in Fig. 3F. Abd. II with m3 and m5 as mac; supplementary chaetae mi, li, lm, ll fan-shape, ml absent, chaeta as fan-shaped, other chaetae as in Fig. 3G. Abd. III with p6 and pm6 as mac; chaeta emp and d3 absent; supplementary chaeta am6 fusiform; chaetae a2, mi, ml, li, ll, lm, im and em fan-shaped, other chaetae as in Fig. 3H. Abd. IV with the chaetae A3–6 as mic, B3–6 as mac, C1p, C2–4 as mic, Sm as mic, T3 as a ciliated mic, T1, T5 and T7 as mic, T6 as mes, D1p as mic and lateral to T3, D2–3 and De3 as mes, E1 and E3 as mac, E2 as mes, F1 and F3 as mes, F2 as mac; supplementary chaetae s absent, a, m, D1, pi and pe with fan-shape and 18 posterior as mac (Fig. 4A and B). Abd. V with the chaetae m2–3 and p3–5 as mac (Fig. 4C).

**Appendages**. Scales absent on Ant. I–IV, apical bulb absent on Ant. IV. Sense organ of Ant. III with two cylindrical chaetae, two blunt guard sensilla and one no modified chaeta. Scales absent on all legs, except on coxae. Trochanteral organ with 7–10 spine-like chaetae. Unguis with four ungual teeth on internal margin: paired BT, unpaired MT and DT unpaired and minute, one basal tooth on external margin and lateral teeth present. Unguiculi truncated with internal margin smooth (Fig. 4D). Tenent hair smooth, apically cuneate and 0.71 as long as outer margin of unguis. Tibiotarsal chaeta smooth and 0.92 as long as unguiculus length. Ratio unguis III: unguiculus III = 2.55:1 in paratype.

Ventral tube with anterior side with 8–10 ciliate chaetae, posterior side 8 ciliate chaetae and lateral side with 3 ciliate chaetae and 9 smooth chaetae. Scales absent on anterior and posterior side.

Furcula with manubrium ventrally with about 10 apical scales; anterior and posterior side without scales; manubrial plate with 5 inner as mac. Mucronal spinelet present (Fig. 4E). Dental tubercle with cylindrical shape and apically domed (Fig. 4F). Pseudopores distribution on dorsal and ventral side of body and appendages as in Fig. 4G (Table 1).

#### Diagnosis

Supplementary chaeta ml absent on Abd. II. Chaeta m7 as mic on Abd. III. Dorsal mac formula A_0_/00/0100+3. Supplementary chaeta s absent on Abd. IV. Mucronal spinelet present.

#### Etymology

This new species is dedicated to the memory of my brother, Deiver Anderson González Montaña (1975-2021).

### Lepidocyrtus rosanae

González-Montaña sp. nov.

CEB7FE8E-5C8B-53C8-8C2D-6B7FA7A1D63C

3386D1EC-F28C-44C4-B330-0D50A639C436

#### Materials

- **Type status:** Holotype. **Occurrence:** recordedBy: L. A. González-Montaña; individualCount: 1; sex: female; occurrenceID: 994C9B68-C97D-5735-8726-9A2EC0BC52E2; **Taxon:** genus: Lepidocyrtus; specificEpithet: rosanae; scientificNameAuthorship: González-Montaña, L. A. ; **Location:** country: Colombia; stateProvince: Casanare; county: Colombia; locality: Sabanalarga; verbatimElevation: 321 m; verbatimLatitude: 4°53’43’’N; verbatimLongitude: 73°01’60’’E; **Event:** year: 2016; month: 12; day: 23
- **Type status:** Paratype. **Occurrence:** recordedBy: L. A. González-Montaña; individualCount: 1; occurrenceID: 28C67773-3B78-54FC-A6FB-6A5223FB1134; **Taxon:** genus: Lepidocyrtus; specificEpithet: rosanae; scientificNameAuthorship: González-Montaña, L. A. ; **Location:** country: Colombia; stateProvince: Casanare; county: Colombia; locality: Sabanalarga; verbatimElevation: 321 m; verbatimLatitude: 4°53’43’’N; verbatimLongitude: 73°01’60’’E; **Event:** year: 2016; month: 12; day: 23
- **Type status:** Paratype. **Occurrence:** recordedBy: L. A. González-Montaña; individualCount: 1; occurrenceID: 70F1EE77-69BE-55CC-AE8A-04C8E133D681; **Taxon:** genus: Lepidocyrtus; specificEpithet: rosanae; scientificNameAuthorship: González-Montaña, L. A. ; **Location:** country: Colombia; stateProvince: Casanare; county: Colombia; locality: Sabanalarga; verbatimElevation: 321 m; verbatimLatitude: 4°53’43’’N; verbatimLongitude: 73°01’60’’E; **Event:** year: 2016; month: 12; day: 23
- **Type status:** Paratype. **Occurrence:** recordedBy: L. A. González-Montaña; individualCount: 1; occurrenceID: 20ED9CD4-F332-5509-83C8-88AEBD287D17; **Taxon:** genus: Lepidocyrtus; specificEpithet: rosanae; scientificNameAuthorship: González-Montaña, L. A. ; **Location:** country: Colombia; stateProvince: Casanare; county: Colombia; locality: Sabanalarga; verbatimElevation: 321 m; verbatimLatitude: 4°53’43’’N; verbatimLongitude: 73°01’60’’E; **Event:** year: 2016; month: 12; day: 23
- **Type status:** Paratype. **Occurrence:** recordedBy: L. A. González-Montaña; individualCount: 1; occurrenceID: 658E232C-CBDB-54E4-82B7-952A8747DD05; **Taxon:** genus: Lepidocyrtus; specificEpithet: rosanae; scientificNameAuthorship: González-Montaña, L. A. ; **Location:** country: Colombia; stateProvince: Casanare; county: Colombia; locality: Sabanalarga; verbatimElevation: 321 m; verbatimLatitude: 4°53’43’’N; verbatimLongitude: 73°01’60’’E; **Event:** year: 2016; month: 12; day: 23
- **Type status:** Paratype. **Occurrence:** recordedBy: L. A. González-Montaña; individualCount: 1; occurrenceID: 65774ECF-5DCD-54B5-9CDC-7D4C0BBF8E5F; **Taxon:** genus: Lepidocyrtus; specificEpithet: rosanae; scientificNameAuthorship: González-Montaña, L. A. ; **Location:** country: Colombia; stateProvince: Casanare; county: Colombia; locality: Sabanalarga; verbatimElevation: 321 m; verbatimLatitude: 4°53’43’’N; verbatimLongitude: 73°01’60’’E; **Event:** year: 2016; month: 12; day: 23
- **Type status:** Paratype. **Occurrence:** recordedBy: L. A. González-Montaña; individualCount: 1; occurrenceID: 1AD28088-42F0-5F96-BCB6-956D6D9DE85C; **Taxon:** genus: Lepidocyrtus; specificEpithet: rosanae; scientificNameAuthorship: González-Montaña, L. A. ; **Location:** country: Colombia; stateProvince: Casanare; county: Colombia; locality: Sabanalarga; verbatimElevation: 321 m; verbatimLatitude: 4°53’43’’N; verbatimLongitude: 73°01’60’’E; **Event:** year: 2016; month: 12; day: 23
- **Type status:** Paratype. **Occurrence:** recordedBy: L. A. González-Montaña; individualCount: 1; occurrenceID: 6F506103-4E90-5B39-9991-022B9DEA50FB; **Taxon:** genus: Lepidocyrtus; specificEpithet: rosanae; scientificNameAuthorship: González-Montaña, L. A. ; **Location:** country: Colombia; stateProvince: Casanare; county: Colombia; locality: Sabanalarga; verbatimElevation: 321 m; verbatimLatitude: 4°53’43’’N; verbatimLongitude: 73°01’60’’E; **Event:** year: 2016; month: 12; day: 23
- **Type status:** Paratype. **Occurrence:** recordedBy: L. A. González-Montaña; individualCount: 1; occurrenceID: DA14E714-200B-5B0F-8032-51BEAD2759E6; **Taxon:** genus: Lepidocyrtus; specificEpithet: rosanae; scientificNameAuthorship: González-Montaña, L. A. ; **Location:** country: Colombia; stateProvince: Casanare; county: Colombia; locality: Sabanalarga; verbatimElevation: 321 m; verbatimLatitude: 4°53’43’’N; verbatimLongitude: 73°01’60’’E; **Event:** year: 2016; month: 12; day: 23
- **Type status:** Paratype. **Occurrence:** recordedBy: L. A. González-Montaña; individualCount: 1; occurrenceID: 75A33898-CF77-548C-9CFB-3AA78BEEEB2A; **Taxon:** genus: Lepidocyrtus; specificEpithet: rosanae; scientificNameAuthorship: González-Montaña, L. A. ; **Location:** country: Colombia; stateProvince: Casanare; county: Colombia; locality: Sabanalarga; verbatimElevation: 321 m; verbatimLatitude: 4°53’43’’N; verbatimLongitude: 73°01’60’’E; **Event:** year: 2016; month: 12; day: 23
- **Type status:** Paratype. **Occurrence:** recordedBy: L. A. González-Montaña; individualCount: 1; occurrenceID: 931EA888-FD3F-5DC1-9B3C-EE7E2581E881; **Taxon:** genus: Lepidocyrtus; specificEpithet: rosanae; scientificNameAuthorship: González-Montaña, L. A. ; **Location:** country: Colombia; stateProvince: Casanare; county: Colombia; locality: Sabanalarga; verbatimElevation: 321 m; verbatimLatitude: 4°53’43’’N; verbatimLongitude: 73°01’60’’E; **Event:** year: 2016; month: 12; day: 23
- **Type status:** Paratype. **Occurrence:** recordedBy: L. A. González-Montaña; individualCount: 1; occurrenceID: 24F09D92-78DF-5C80-AB5A-8DFBDB910A26; **Taxon:** genus: Lepidocyrtus; specificEpithet: rosanae; scientificNameAuthorship: González-Montaña, L. A. ; **Location:** country: Colombia; stateProvince: Casanare; county: Colombia; locality: Sabanalarga; verbatimElevation: 321 m; verbatimLatitude: 4°53’43’’N; verbatimLongitude: 73°01’60’’E; **Event:** year: 2016; month: 12; day: 23

#### Description

Holotype body mean length without appendages 1.86 mm (n = 12). Th. II protruding over head. Holotype with Ant. III–IV distally pigmented. Head with dark blue spot on dorso-anterior edge and lateral stripe dark blue; Th. III–Abd. II with a lateral dark blue spot; Abd. IV with postero-lateral spot dark blue (Fig. 5A). Body colour variation observed where postero-lateral spot dark blue is absent on Abd. IV (*Fig. 5* B).

**Head**. Dorsal cephalic chaetae with the chaeta A0 not recognised, A1 as mes, A2 as mes, A2a as mac, chaetae An on head between 12–17, other chaetae as in Fig. 6A. Interocular chaetae q, s, p, r and t present and intraocular scales absent (Fig. 6A).

Clypeal chaetae ciliate. Prelabral chaetae ciliated. Labral chaetae a1 branched apically, bi- or trifurcate (Fig. 6B), a2, m0–2, p0–2 smooth, subequal, four labral papillae with inner papillae apically rounded and outer apically truncated.

Maxillary outer lobe with apical appendage and basal chaeta smooth; appendages absent on sublobal plate (Fig. 6C).

Labial chaetae a1–5 smooth and posterior row with formula M_2_rEL_1_L_2_, chaetae ciliated with same length and r as spine. Labial papillae A–E with apparently 0, 4, 0, 2, 4 guard appendages, labial papilla E with lateral process with finger-shape and not reaching tip of papilla and labial papilla H with two guard appendages (not drawn) (Fig. 6D-F). Postlabial chaetotaxy with all chaetae ciliated, five chaetae along ventral groove and two rows with 3 (G1–G3) and 1 (X3).

**Body**. Dorsal macrochaetae formula 00/0100+2. Th. II as in Fig. 6G. Th. III as in Fig. 6H and sens al not observed. Chaetotaxy of Abd. I as Fig. 6I. Abd. II with m3 and m5 as mac; supplementary chaetae ml, li, lm, ll fan-shape, mi absent, chaeta a2 fan-shaped; sens as absent (Fig. 7A). Abd. III with chaetae a6–7 as fan-shape, supplementary chaetae a2, ml, mi, ll, lm, li, am6, emp, em, im fan-shape; sens as and ms absent (Fig. 7B). Abd. IV with A4–6, B3–4 as mic, B5–6 as mac, C1–4 as mic, C1p as fan-shape, Sm as mic, T3 as a smooth mic, T1, T5–6, T7 as mes, D1p mic and laterad to chaeta T3, D2 mes, D3 mac, De1 as mic, E1 mes, E2–4 as mac, E4p mes, F1 and F3 as mac, F2 mes, F2p as mic; supplementary chaetae a, m, s, D1, pe, pi fan-shape and 21 posterior mes, sens al and ps present (Fig. 7C). Abd. V with m2–3, p4–5 as mac and p3 as mes (Fig. 7D).

**Appendages**. Scales absent on Ant. I–IV, apical bulb unilobed on Ant. IV. Sense organ of Ant. III with two cylindrical chaetae. Scales absent on all legs, except on coxae. Trochanteral organ with 9–15 spine-like chaetae. Unguis with four ungual teeth on internal margin: paired BT, unpaired MT and DT unpaired, one basal tooth on external margin and lateral teeth present. Unguiculi truncated and external margin serrated (Fig. 7E). Tenent hair smooth, apically cuneate and subequal to external edge of unguis. Tibiotarsal chaeta smooth chaeta 0.92 as long as unguiculus length. Ratio unguis III: unguiculus III = 1.422:1 in paratype.

Ventral tube with Anterior side with 14–15 chaetae and 8–10 scales on anterior side (not drawn), posterior side with 5 ciliated chaetae and lateral side with 6 ciliated chaetae and 7 smooth chaetae.

Furcula with manubrium ventrally with about 15 apical scales; anterior and posterior side without scales; manubrial plate with 4 inner as mac. Mucronal spinelet present. Dental tubercle with conical shape, small and apically domed (Fig. 7F). Pseudopores distribution on dorsal and ventral side of body and appendages as in Fig. 7G (Table 1).

#### Diagnosis

Scales present on anterior side of collophore. Labral chaetae a1 apically branched. Appendages absent on sublobal plate. Sens as absent on Abd. II, sens as and ms absent on Abd. III. Supplementary chaetae s on Abd. IV present. Mucronal spinelet present.

#### Etymology

The new species is named in honour to my mother, María Rosana Montaña Lemus. The species epithet is a noun in apposition.

## Discussion

Lepidocyrtus (S.) deiverandersoni **sp. nov**. is placed in the subgenus *Setogaster* Salmon 1951, based on absence of apical bulb on Abd. IV and presence of mucronal spinelet (*sensu* Cipola et al. (2019)), while other characters, such as scales on appendages or rounded dental tubercle, could be variable and shared with other subgenera (Mateos and Greenslade 2015, Cipola et al. 2019). Lepidocyrtus (S.) deiverandersoni **sp. nov**. resembles *L. marimutti* Thibaud & Najt, 1987 and *L. asensillatus* Cipola & Bellini, 2026 by inner mac on Abd. IV. The new species differs from the first species chaeta m7 by mic on Abd. III and supplementary chaeta li and mi present on Abd. III, while some remarkable differences with the second species are one labial chaeta M_1_ absent, sens as present on Abd. II–III and chaeta m3 as mac.

In addition, L. (S.) deiverandersoni **sp. nov**. resembles the Neotropical species *L. dispar* Mari Mutt, 1986, *L. griseolus* Mari Mutt, 1986, *L. maldonadoi* Mari Mutt, 1986, *L. balteatus* Mari Mutt, 1983 and *L. distinctus* Mari Mutt, 1986 by body colour pattern. Lepidocyrtus (S.) deiverandersoni **sp. nov**. differs from *L. dispar* and *L. griseolus* by supplementary chaeta s absent and chaeta B4 as mac on Abd. IV, while *L. griseolus* shows cephalic dorsal mac; from *L. maldonadoi* and *L. balteatus* by apical bulb absent and chaeta B4 and B6 as mac and, with respect to *L. distinctus*, by the shape of supplementary chaetae, which are subulate in this species.

Amongst Neotropical species, *L. rosanae* **sp. nov**. has a distinctive combination of taxonomic characters, including labral chaetae a1 apically branched, appendages absent on sublobal plate, sens as absent o Abd. II and sens ps and ms absent on Abd. II–III, also absent in *L.* asensillatus (Cipola and Bellini 2026) and scales present on anterior side of collophore. *Lepidocyrtus rosanae* **sp. nov**. resembles *L. caprilesi* Wray, 1953, and *L. paracaprilesi* Mari Mutt, 1988 by the Th. II protruding over head, scales present on collophore, but the labial chaetae M_1_ and M_3_ are absent in the new species. Likewise, *L. rosanae* **sp. nov**. resembles *L. dispar* and *L. biphasis* Mari Mutt, 1986 by the body colour pattern and two inner mac on Abd. IV, but differs from *L. dispar* by the apical bulb present on Ant. IV and from *L. biphasis* by sens as and ms absent on Abd. II–III.

*Lepidocyrtus rosanae* **sp. nov**. shows mucronal spinelet suggesting its position in the *Setogaster*, but the presence of apical bulb on Ant. IV and presence of scales on anterior side of ventral tube, casts doubt on its position. The last characters are shown by *Acrocyrtus* although the dental tubercle acute is absent. The subgeneric position of *L. rosanae* **sp. nov**. is uncertain.

The new species are part of group, characterised by labial chaeta M_1_ absent (Table 2), integrated by the most Neotropical *Lepidocyrtus* (see Mari Mutt (1983), Mari Mutt (1986), Mari Mutt (1987)), the Neartic species *L. floridensis* Sneider, 1967, *L. bobwoodruffi* Soto-Adames, Jenks, & Daly, 2025, *L. cyaneus* Tullberg, 1871, *L. fernandi* Christiansen & Bellinger, 1998, the European species *L. bicoloris* Mateos, 2012, *L. selvaticus* Arbea & Ariza, 2007, *L. lusitanicus* Gama, 1964, *L. fimetarius* Gisin, 1964, *L. lanuginosus* Tullberg, 1871 and the Asian species *L. filamentosus* Yoshii, 1982 and *L. gumgum* Yoshii, 1982, although the labial chaeta M_1_ could be absent/present in *L. bicoloris*, *L. lusitanicus*, *L. fimetarius*, *L. lanuginosus* and *L. cyaneus* (Snider 1967, Yoshii 1982,Mari Mutt 1986, Christiansen and Bellinger 1998, Mateos 2008a, Mateos 2008b, Mateos 2012). Likewise, other characters, such as the cephalic dorsal mac and scales absent on appendages, are observed in the most Neotropical species with one labial chaeta M_1_.

The characters mentioned above suggest relationships between Neotropical species and the Holartic and Indo-Malay species. Soto-Adames et al. (2025) pointed out the relationship between *L. floridensis* and Neotropical species by dorsal cephalic mac being absent, chaeta a3 internal to a2 on Abd. II and the presence of dental tubercle. Following this reasoning, *L. fimetarius* (individuals where labial chaeta M_1_ is absent) is similar to *L. pseudofimetarius*, *L. ramosi* and *L. nilatus*, not only by labial chaeta M_1_ absent, but also by the dorsal cephalic mac being absent and inner mac on Abd. IV, suggesting relationships between Neotropical and Paleartic species.

The relationships between Neotropical species and the Indo-Malay species have not been explored, although *L. wanningensis*, described by Ma (2019) from south of China and *L. olena* from Pacific Islands, resembles the Neotropical species *L. nigrosetosus* (Bernard et al. 2015, Enguídanos et al. 2024), while the absence of labial chaeta M_1_ in *L. gumgum* and *L. filamentosus* suggests relationships with Neotropical species. On which characters could be used to delimit the subgenera in *Lepidocyrtus* is continuously addressed in the taxonomic literature, which is still in conflict for Neotropical species. In the phylogenetic framework, testing should be undertaken, for example, to determine the usefulness of labial chaetae in supporting natural groups. Additionally, biogeographic patterns shown by *Lepidocyrtus* are interesting, such as the possible relationship between Neotropical species and Indo-Malay species; however, these patterns reflect a long tradition in the taxonomy of genus, requiring the exploration of other regions as well as the Andean Region, where the richness of *Lepidocyrtus* is probably high.

## Supplementary Material

XML Treatment for Lepidocyrtus (Setogaster) deiverandersoni

XML Treatment for Lepidocyrtus rosanae

8FCEC142-E850-577A-8122-CD714C0833F5 10.3897/BDJ.14.e207059.suppl1 Supplementary material 1 Anatomical and phenotypic terms used in the description of *Lepidocyrtus deiverandersoni* sp. nov. and *Lepidocyrtus rosanae* sp. nov Data type Antomical ontology Brief description File contains a list of anatomical and phenotypic terms used in the description of new species and is available in the main anatomical ontologies "Collembola Anatomy Ontology" and "Phenotype And Trait Ontology". Due to restrictions in the building of CLAO, such as redundant labels, some terms are available only as synonyms. Currently, other terms and definitions are added. File: oo_1701207.xlsx https://binary.pensoft.net/file/1701207 González-Montaña, L. A.

## Figures and Tables

**Figure 1.**
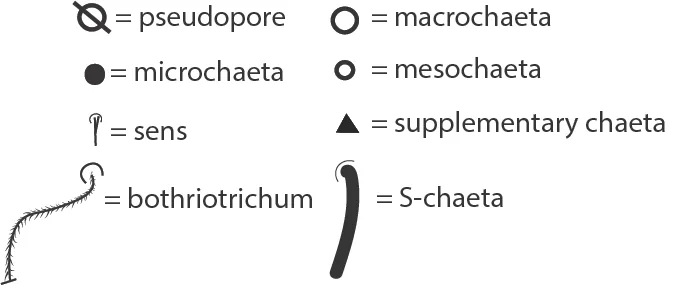
Symbols and chaeta types used in the illustrations. The size of the chaetae sockets is proportional to their size.

**Figure 2.**
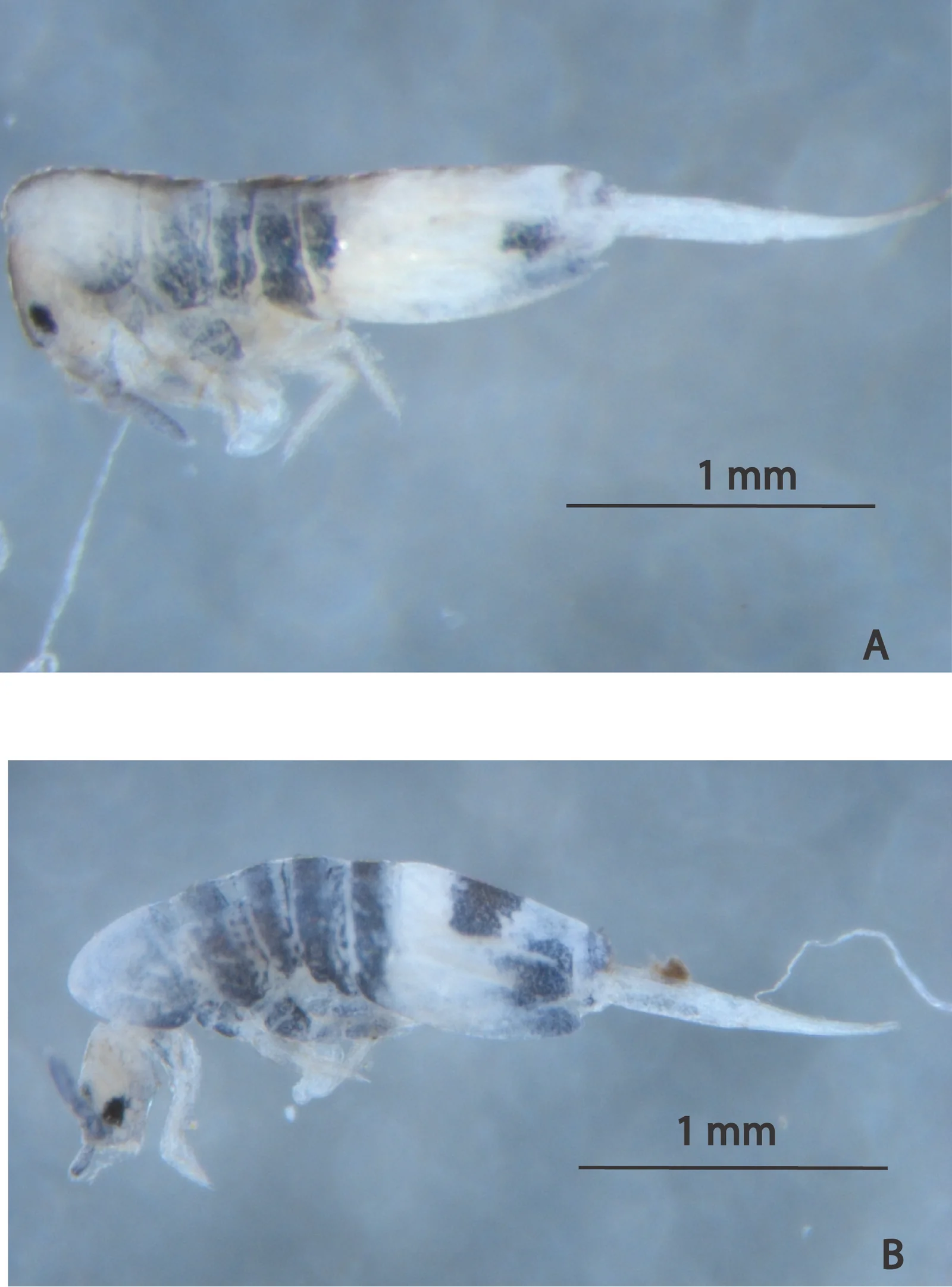
Habitus and variation of the colour pattern of L. (S.) deiverandersoni **sp. nov. A** habitus holotype; **B** variation.

**Figure 3.**
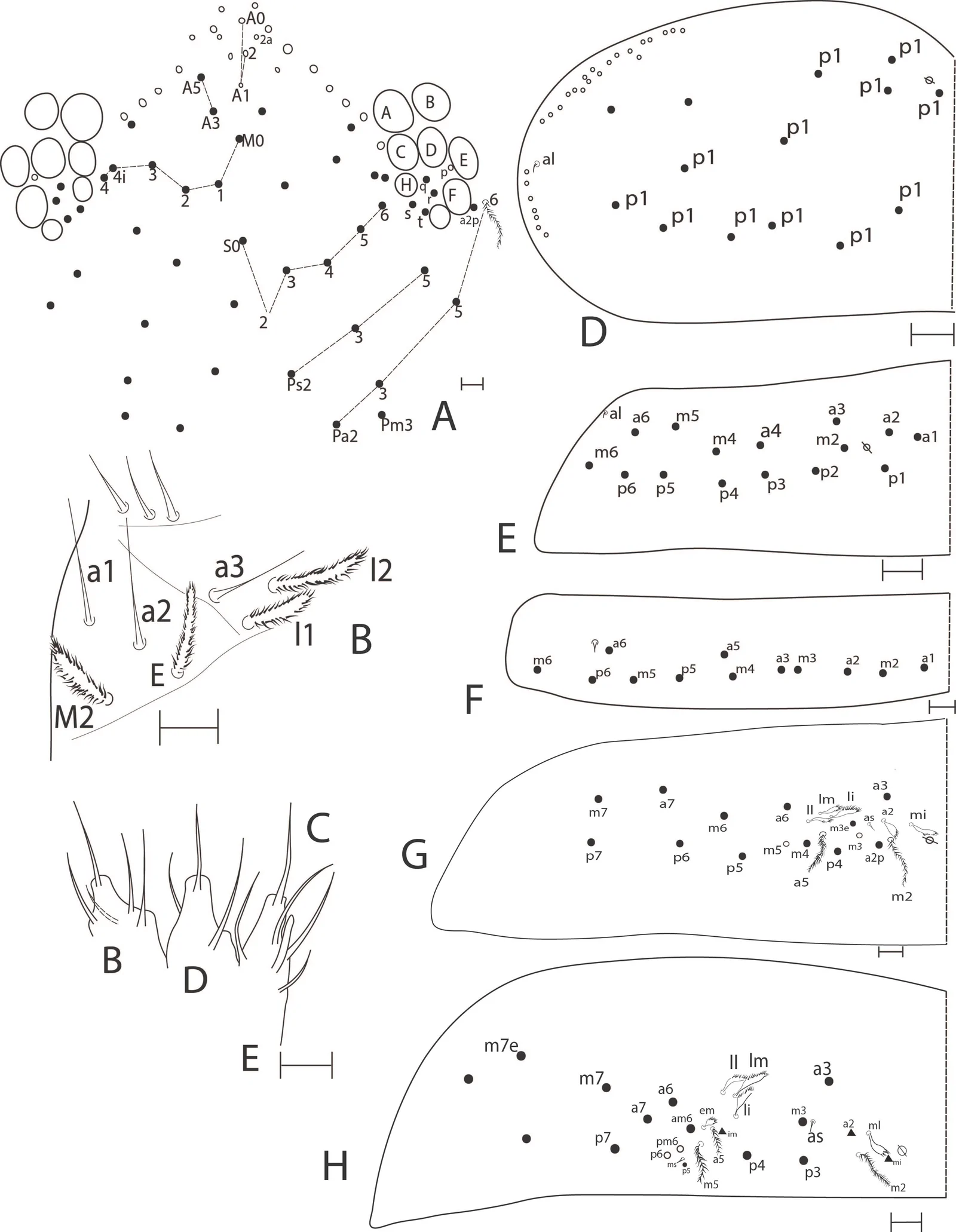
Lepidocyrtus (S.) deiverandersoni **sp. nov. A** Head dorsal chaetotaxy; **B** labial chaetotaxy; **C** labial palp; **D** Th. II chaetotaxy; **E** Th. III chaetotaxy; **F** Abd. I chaetotaxy; **G** Abd. II chaetotaxy; **H** Abd. III chaetotaxy.

**Figure 4.**
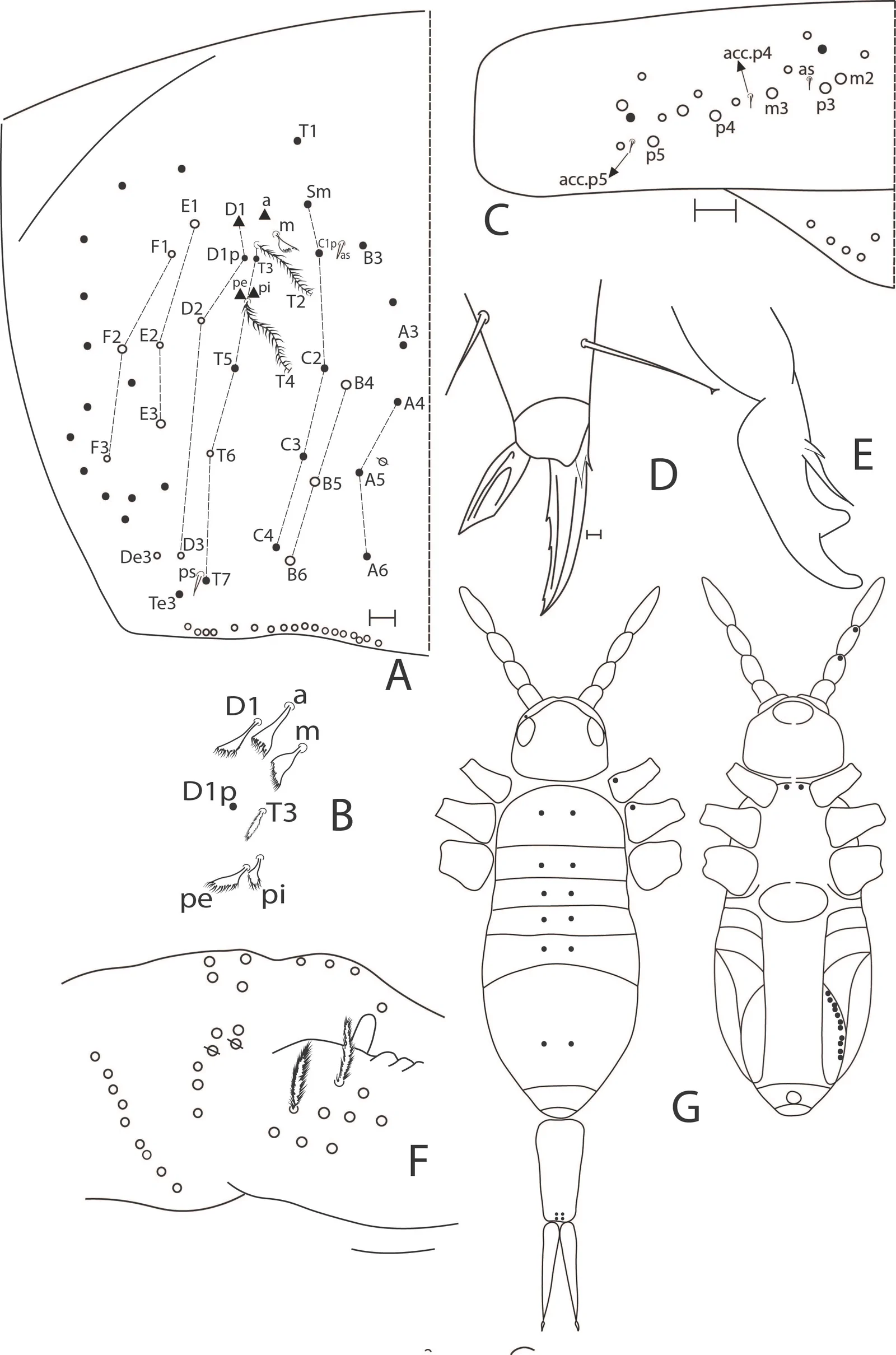
Lepidocyrtus (S.) deiverandersoni **sp. nov. A** Abd. IV chaetotaxy; **B** Detail of bothriotrical complex of the Abd. IV; **C** Abd. V–VI chaetotaxy; **D** unguis and unguiculus of third leg; **E** mucro; **F** manubrial plate; **G** Distribution of pseudopores on dorsal and ventral side.

**Figure 5.**
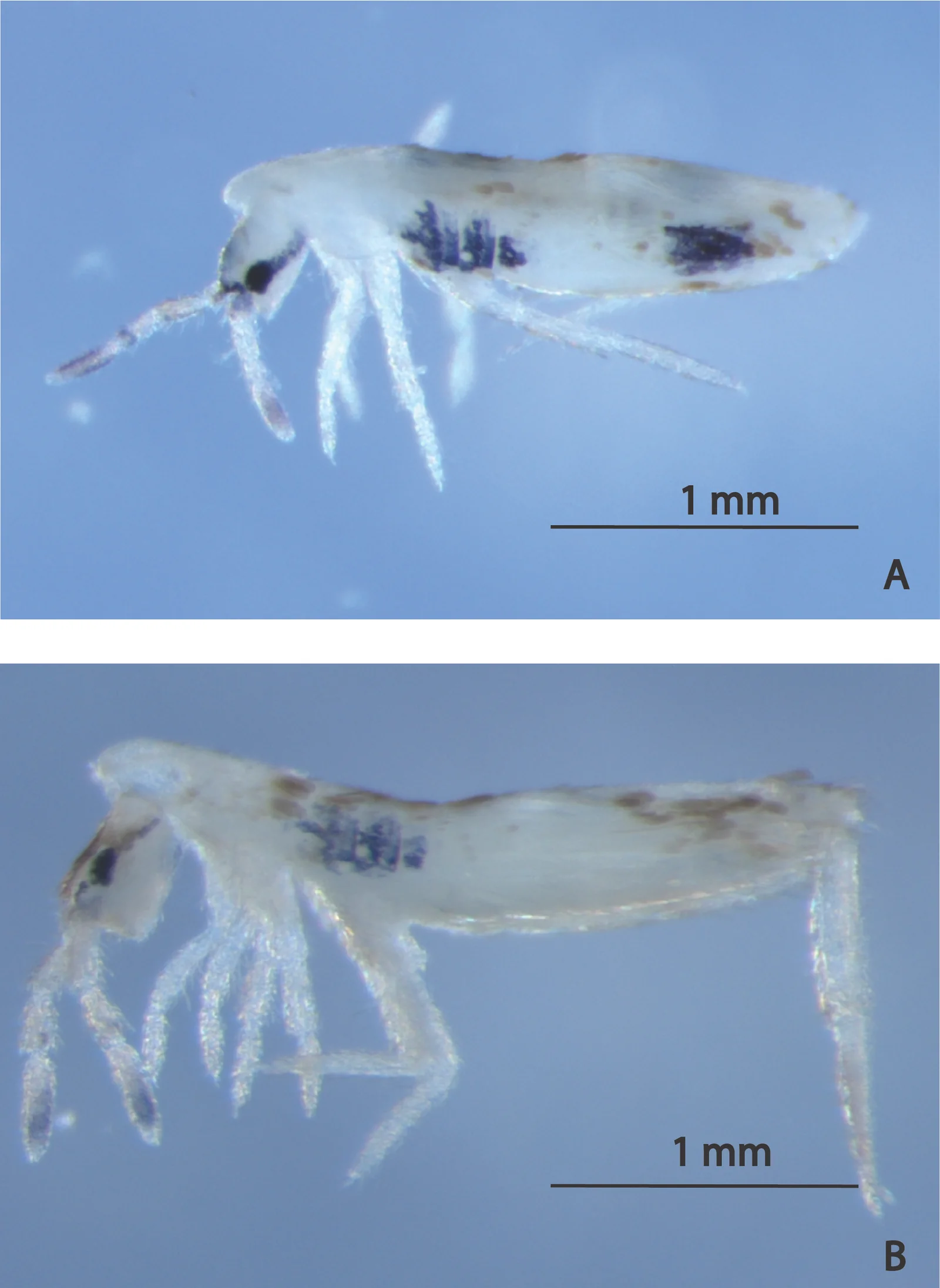
Habitus and variation of the colour pattern of *L. rosanae* **sp. nov. A** Holotype; **B** variation.

**Figure 6.**
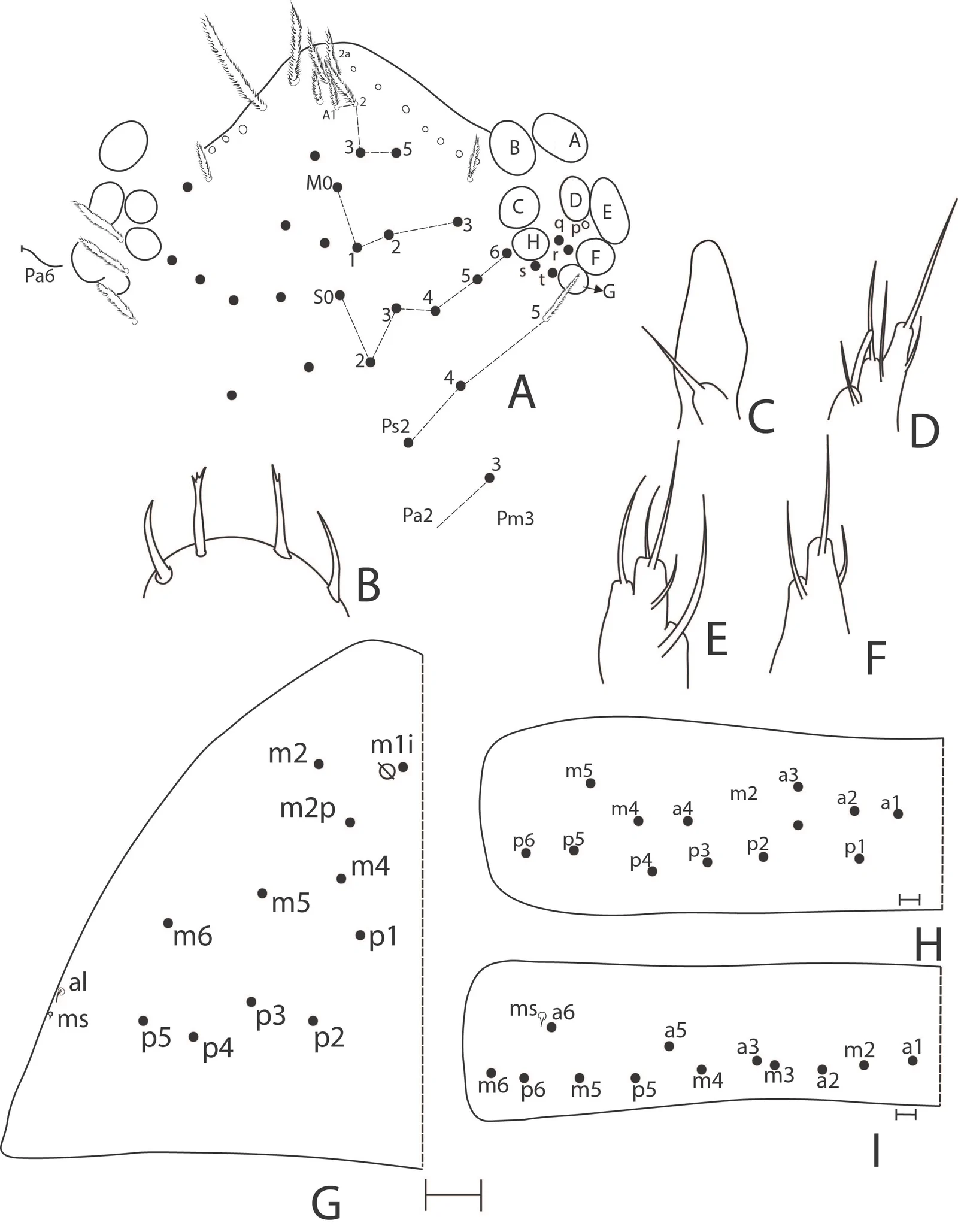
*Lepidocyrtus rosanae* **sp. nov. A** Head dorsal chaetotaxy; **B** Detail of labral chaetae row a, with chaetae a1 branched apically; **C** Detail of sublobal plate; **D** labial papillae E; **E** labial papillae B; **F** labial papillae D; **G** Th. II chaetotaxy; **H** Th. III chaetotaxy; **I** Abd. I chaetotaxy.

**Figure 7.**
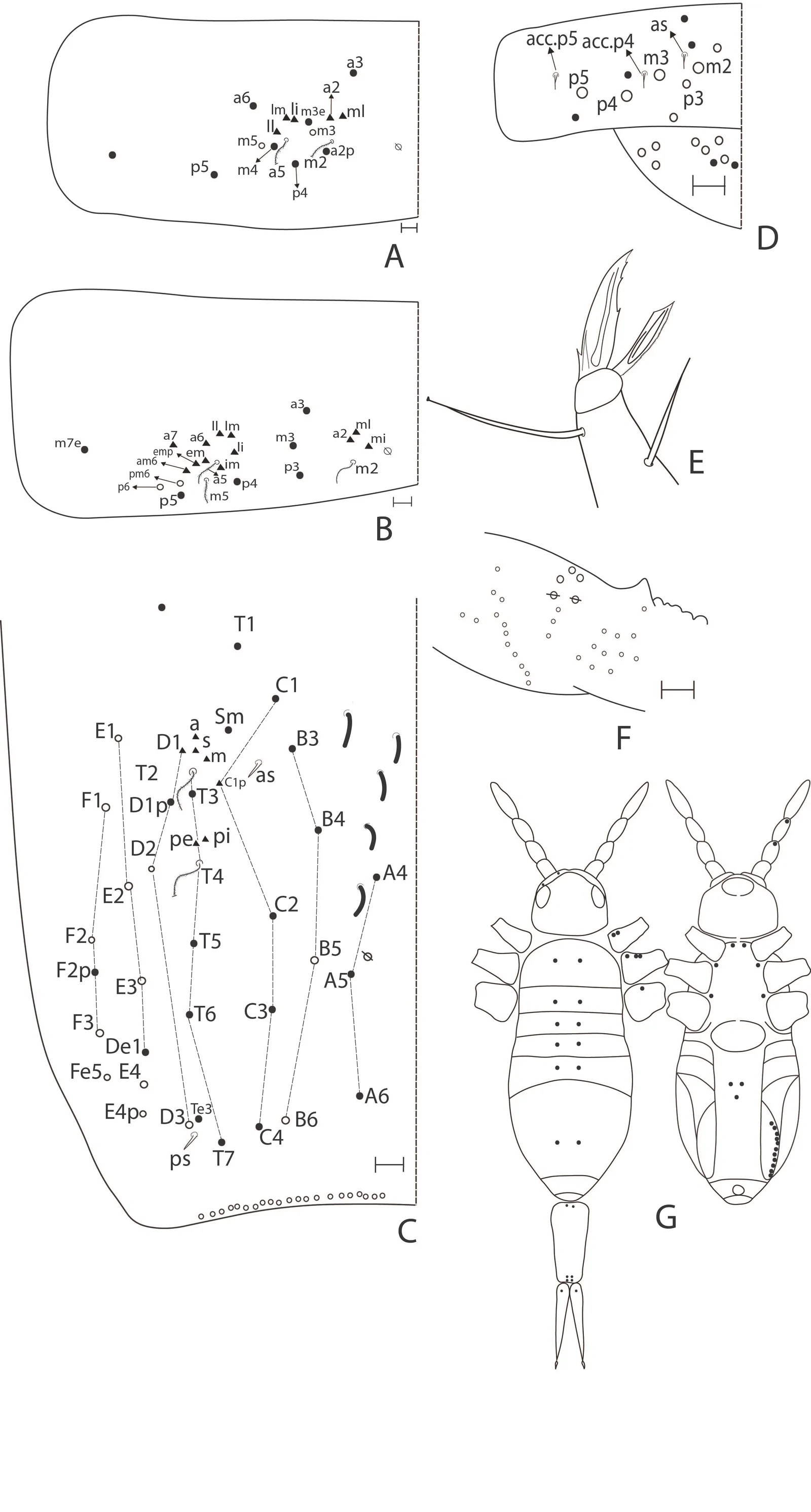
*Lepidocyrtus rosanae* **sp. nov. A** Abd. II chaetotaxy; **B** Abd. III chaetotaxy; **C** Abd. IV chaetotaxy; D Abd. V–VI chaetotaxy; unguis and unguiculus of third leg; F manubrial plate; G distribution of pseudopores on dorsal and ventral side.

**Table 1.** Distribution of pseudopores in L. (S.) deiverandersoni **sp. nov**. and *L. rosanae* **sp. nov**.

Body region	Position	L. (S.) deiverandersoni**sp. nov**.	*L. rosanae***sp. nov**.
Head dorsal	Pre-ocular	1	1
	Clypeus	0	1
Body dorsal	Th.II	1	1
	Th.III	1	1
	Abd.I	1	1
	Abd.II	1	1
	Abd.III	1	1
	Abd.IV	1	1
	Abd.IV	1	1
Body ventral	Th.I	1	1
	Th.II	0	1
	Th.III	0	1
	Abd.I–Collophore base anterior	0	0
	Abd.I–Collophore base posterior	0	0
	Abd.II	0	1
	Abd.III–post-retinaculum	0	1
	Abd.IV	0	0
	Abd.V–pre-genital plate	0	0
Body lateral	BP4	11	11
Antenae ventral	Ant.I	0	0
	Ant.II	0	1
	Ant.III	0	1
Legs	Cx.I	1	2
	Cx.II	1	3
	Cx.III	1	1
Furcula dorsal	Manubrial base	0	0
	Manubrial plate	2	2
	Dens	0	1

**Table 2.** Comparative table of species with one labial M. *1 L. deiverandersoni* **sp. nov**.; 2. *L. rosanae* **sp. nov**.; 3. *L. marimutti*; 4 *L. dispar; 5 L. griseolus; 6 L. maldonadoi; 7 L. distinctus*; 8 *L. biphasis*; 9 *L. balteatus*; 10 L. diminutus; 11 *L. ianthinus*; 12 *L. lepargus*; 13 *L. ramosi*; 14 *L. vireticulus*; 15 *L. nilatus*; 16 L. pseudofimetarius; 17 *L. fimetarius*; 18 *L. labursbichaetus*; 19 *L. floridensis*; 20 *L. bobwoodruffi; 21 L. cyaneus; 22 L. fernandi; 23 L. bicoloris; 24 L. selvaticus; 25 L. lusitanicus; 26 L. filamentosus; 27 L. gumgum*. (#). This character interpreted as tiny spines by Mari Mutt (1986), (*) species with labial chaeta M_1_ is variable. References: Yoshii (1982), Gruia (1983), Mari Mutt (1983), Mari Mutt (1986), Mari Mutt (1987), Wang et al. (2003) Mateos (2008a), Mateos (2008b), Mateos (2012), Soto-Adames et al. (2025), Cipola and Bellini (2026).

Character	1	2	3	4	5	6	7	8	9	10	11	12	13	14	15	16	17	18	19	20	21*	22	23*	24	25*	26	27
apical bulb on Ant. IV	−	+	−	−	+	+	+	+	+	+	−	+	+	−	+	+	+	+/-	−	−	−	−	−	−	+	−	+
scales on appendages	−	+	−	−	−	−	−	−	−	−	−	−	−	−	−	−	−	−	−	−	−	−	−	−	−	−	−
suppl. chaeta mi on Abd. III	+	+	−	+	+	+	+	+	+	+	+	+	+	+	+	+	+	−	+	+	+	?	+	+	+	?	?
suppl. chaeta li on Abd. III	+	+	−	+	+	+	+	+	+	+	+	+	+	+	+	+	+	+	+	+	+	+	+	+	+	?	?
Head dorsal pentangle	−	−	−	−	−	−	−	−	−	−	−	−	−	+	−	−	−	−	−	−	+	+	+	+	+	?	?
suppl. chaeta s	−	−	−	+	+	−	−	+	+	−	−	−	−	+	−	−	−	−	−	−	−	?	−	−	−	?	−
chaeta Sm	mic	mic	mic	mic	mic	mic	mic	mic	mic	mic	mic	mic	mic	mac	mic	mic	mic	mic	mic	mic	mic	mac	mic	mac	mac	?	?
chaeta B4 on Abd. IV	mac	mic	mac	mic	mic	mic	mac	mic	mic	mic	mic	mic	mac	mic	mac	mac	mac	mac	mic	mic	mic	mic	mic	mac	mac	?	?
chaeta B6 on Abd. IV	mac	mac	mac	mac	mac	mes	mic	mac	mic	mac	mac	mac	mic	mac	mic	mic	mic	mic	mac	mac	mac	mac	mac	mac	mac	?	?
sens as and ps on Abd. III	+	−	+	+	+	+	+	+	+	+	+	+	+	+	+	+	+	+	+	+	+	+	+	+	+	?	?
mucronal spinelet	+	+	+	#	#	#	#	#	#	#	#	#	#	#	−	−	−	−	+	−	−	−	−	−	−	+	?
